# AMR-Diag: Neural network based genotype-to-phenotype prediction of resistance towards β-lactams in *Escherichia coli* and *Klebsiella pneumoniae*

**DOI:** 10.1016/j.csbj.2021.03.027

**Published:** 2021-03-29

**Authors:** Ekaterina Avershina, Priyanka Sharma, Arne M. Taxt, Harpreet Singh, Stephan A. Frye, Kolin Paul, Arti Kapil, Umaer Naseer, Punit Kaur, Rafi Ahmad

**Affiliations:** a Department of Biotechnology, Inland Norway University of Applied Sciences, Holsetgata 22, 2317 Hamar, Norway; b Department of Biophysics, All India Institute of Medical Sciences, New Delhi, India; c Department of Microbiology, Division of Laboratory Medicine, Oslo University Hospital, PB 4956, Nydalen, 0424 Oslo, Norway; d Informatics, System and Research Management, Indian Council of Medical Research, New Delhi, India; e Department of Computer Science & Engineering, IIT Delhi, New Delhi, India; f Department of Microbiology, All India Institute of Medical Sciences, New Delhi, India; g Department of Zoonotic, Food- and Waterborne Infections, 0213 Oslo, Norwegian Institute of Public Health, Oslo, Norway; h Institute of Clinical Medicine, Faculty of Health Sciences, UiT - The Arctic University of Norway, Hansine Hansens veg 18, 9019 Tromsø, Norway

**Keywords:** Antibiotic resistance, Genotype to phenotype, Machine learning, Neural networks, Extended spectrum β-lactamases, Colistin, *E. coli*, *K. pneumoniae*, Carbapenemases, Antibiotic susceptibility testing, Rapid diagnostics, Bacterial infection

## Abstract

Antibiotic resistance poses a major threat to public health. More effective ways of the antibiotic prescription are needed to delay the spread of antibiotic resistance. Employment of sequencing technologies coupled with the use of trained neural network algorithms for genotype-to-phenotype prediction will reduce the time needed for antibiotic susceptibility profile identification from days to hours.

In this work, we have sequenced and phenotypically characterized 171 clinical isolates of *Escherichia coli *and *Klebsiella pneumoniae* from Norway and India. Based on the data, we have created neural networks to predict susceptibility for ampicillin, 3rd generation cephalosporins and carbapenems. All networks were trained on unassembled data, enabling prediction within minutes after the sequencing information becomes available. Moreover, they can be used both on Illumina and MinION generated data and do not require high genome coverage for phenotype prediction. We cross-checked our networks with previously published algorithms for genotype-to-phenotype prediction and their corresponding datasets. Besides, we also created an ensemble of networks trained on different datasets, which improved the cross-dataset prediction compared to a single network.

Additionally, we have used data from direct sequencing of spiked blood cultures and found that AMR-Diag networks, coupled with MinION sequencing, can predict bacterial species, resistome, and phenotype as fast as 1–8 h from the sequencing start. To our knowledge, this is the first study for genotype-to-phenotype prediction: (1) employing a neural network method; (2) using data from more than one sequencing platform; and (3) utilizing sequence data from spiked blood cultures.

## Graphical abstract

**Figure:**
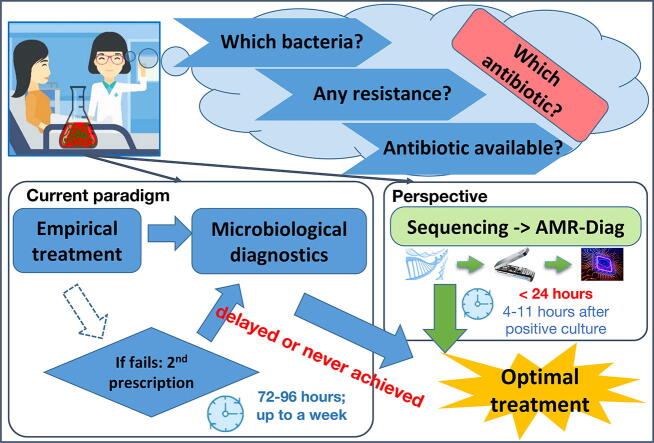

## 1 Introduction

Around 33,000 European inhabitants die from infections caused by bacteria resistant to antibiotics annually [1], and we might witness a tenfold increase in the number of deaths by 2050, given the current state of AMR development [2]. There is a growing concern regarding the dearth of new antibiotic scaffolds in the drug discovery pipeline, especially for multidrug-resistant bacteria that produce Extended Spectrum β-Lactamases (ESBLs) and carbapenemases. β-lactam antibiotics represent the most common drug class of antimicrobial drugs with broad clinical indications. This class of antibiotics makes up 65% of the total antibiotics market [3]. In 2017, WHO published a list of pathogens for which urgent global action is needed [4]. This list includes 3rd generation cephalosporin and carbapenem-resistant Enterobacteriaceae, which are among the priority 1 section of the WHO pathogen list. The β-lactamases produced by the Enterobacteriaceae family, particularly *Klebsiella pneumoniae* and *Escherichia coli,* are hydrolytic enzymes that confer bacterial resistance to β-lactam antibiotics, such as the penicillin, cephalosporin, and carbapenem families that are common antimicrobial drugs used all around the world [4], [5].

A cornerstone of optimal antibiotic use is an informed and rapid susceptibility profile of the pathogen. Traditional microbial diagnostics may require 3–4 days to a week for bacterial culture, isolation of infectious agents, and antibiotic susceptibility testing (AST) [6]. Therefore doctors are often forced to prescribe antibiotics based on empirical guidelines and local epidemiological data. The mortality risk doubles with a 24-hour delay in providing appropriate antibiotics in bacteremia cases [7].

One way to speed up microbial diagnostics is the fast sequencing of the infectious agent and prediction of its phenotype based on whole-genome sequencing (WGS) data. Based on the review of the European Committee on Antimicrobial Susceptibility Testing (EUCAST), the published evidence for using WGS as a tool to infer antimicrobial susceptibility accurately is currently either poor or non-existent, and the evidence/knowledge base requires significant expansion [8]. Experts have proposed that the primary comparators for assessing genotypic-phenotypic concordance from WGS data should be changed to epidemiological cut-off values (ECOFF) to improve differentiation of wild-type from non-wild-type isolates (harboring an acquired resistance). Therefore, clinical breakpoints should be used as a secondary comparator, ideally using the same data sets as used for ECOFF-based assessments. This assessment will reveal whether genome-based predictions could also be used to guide clinical decision making [8].

The very limited availability of rapid, easy to use, and scalable methods to interpret the WGS data for clinical purposes is challenging. Data-driven machine learning (ML) is a promising approach for this kind of data analysis. Some ML-based algorithms for phenotype [9], AMR gene content [10], or MIC prediction [11] have been proposed in recent times (see a detailed review in [12]). Both assembled genomes [10], [11] and raw unassembled sequencing data have been evaluated [11]. Also, k-mer profiles have been used to predict resistance [11], [13].

Previous studies have shown that the trained model often requires only a few genomic features, making it unnecessary to analyze the complete genome for generating predictions for new samples [14], [15]. Additional issues that need to be considered when developing a reliable and useful prediction model include the fact that genotypes are often geographically clustered. This means that if a prediction model is trained on data from one country, the model might not be generalizable to data from another country [16]. Data from multiple countries potentially must be used in any proposed model. India and Norway have a very different spectrum of AMR profiles. Norway has very low levels of AMR, whereas India has relatively higher levels [17]. While most Norwegian isolates are sensitive to antibiotics, India has mostly resistant isolates. Therefore, our collection of isolates gives us a good spectrum of sensitive and resistant strains for building a robust prediction model. Moreover, all the previously published studies have used Illumina sequencing technology. Illumina sequencing is not clinically time-efficient, and recent studies have shown that Oxford Nanopore Technology’s (ONT) MinION could potentially be used for point of care sequencing and, thus, may become a basis for WGS-based diagnostic strategies [6], [18].

This paper presents an assembly-free neural network based method for predicting phenotypic resistance of *E. coli* and *K. pneumoniae* towards 3rd gen. cephalosporins and carbapenems from WGS data. The current model offers a prediction for six antibiotics. Overall, network accuracy ranges between 94% and 100%, and it is independent of the sequencing platform used to generate the data. We have also compared our networks with other previously published ML algorithms for genotype-to-phenotype prediction. To the best of our knowledge, this is the first study for (1) employing a neural network that uses (2) data from more than one sequencing platform; and (3) additionally testing spiked blood cultures for prediction of phenotype from genotype.

## 2 Materials and methods

### 2.1 Bacterial strains

A total of 171 bacterial isolates from human urine, blood, sputum, pus, ascitic, and cystic fluid samples isolated in Norway and India were used in this study. Norwegian isolates were collected from October 2015 to December 2019, whereas Indian strains were collected from December 2017 to August 2019. Strains were collected and phenotypically characterized at Oslo University Hospital (Oslo, Norway) and All India Institute of Medical Science (New Delhi, India). This included 90 *E. coli* and 76 *K. pneumoniae* isolates. Five sequenced isolates were later found to be wrongly assigned as *E. coli* and *K. pneumoniae* at the start of the study. Two of these belonged to *Enterobacter bugadensis* and *Enterobacter cloacae*, and the other three isolates were found to be *K. quasipneumoniae.* All these five isolates were excluded from our training dataset.

In Norway, minimum inhibitory concentration (MIC) (performed at Norwegian Institute of Public Health, Oslo) for ampicillin, ceftazidime, cefotaxime, meropenem and imipenem was determined by broth microdilution using Sensititre surveillance EUVSEC™ 96 well plates (ThermoFisher). Plates were inoculated using the Sensititre AutoInoculator™ (AIM, V3020, Sensititre), incubated for 18–24 h, and subsequently read both with the naked eye using the Sensititre Manual Viewbox™ (V4007, Sensititre) and using the Sensititre Vizion Digital MIC Viewing System™ (V2021, Sensititre). *E. coli* ATCC 25922 and *E. coli* IP2.1 were used as quality strains for *E. coli* and *K. pneumoniae* isolates, respectively. Quality strains were tested in triplicates, and the samples were analyzed once and repeated in case of uncertain results. In India, Mueller Hinton agar medium (BD Difco, Sparks, MD, USA) was inoculated with bacterial inoculum matching a turbidity of 0.5 McFarland standard and then incubated for 18–24 h at 37 °C for antibiotic susceptibility determination. MIC for ampicillin, ceftazidime, cefotaxime, meropenem, imipenem was determined by MIC gradient strips (Himedia Laboratories Ltd, Mumbai, India) according to the manufacturer’s instructions. Antimicrobial susceptibility towards ertapenem was determined by Kirby-Bauer disk diffusion test as per CLSI guidelines using 10 μg antibiotic disks (Himedia Laboratories Ltd, Mumbai, India) [19]. *E. coli* ATCC 25,922 was used as a quality control strain.

Epidemiological cut-off values (ECOFF) for wild-type (WT) vs. non-wild-type (NWT) differentiation was taken from the European Committee on Antimicrobial Susceptibility Testing MIC distribution website [20] (accessed on February 2020). Ertapenem ECOFF value was taken from the Rationale for the EUCAST Clinical Breakpoints v 1.3 (June 2009). Additionally, isolates were also classified as susceptible (S), susceptible to increased exposure (I), and resistant (R) according to EUCAST breakpoints v11.0 (December 2020).

### 2.2 DNA isolation and sequencing

DNA for Illumina sequencing was prepared using the CTAB method described elsewhere [21]. Genomic DNA was isolated from freshly grown strains using QIAamp DNA minikit (Qiagen, Germany) following the manufacturer’s instructions and was quantified using Qubit fluorometer (Life Technologies, USA). Sequencing libraries were prepped using Illumina Nextera XT DNA sample preparation kit (Illumina, USA) and were sequenced on a MiSeq Illumina platform using MiSeq v3 chemistry. Strains from Norway were sequenced at the Norwegian Sequencing Centre, whereas Indian isolates were sequenced at All India Institute of Medical Sciences. Output data files were de-multiplexed and transformed into *fastq* files with Casava v.1.8.2 (Illumina Inc, USA).

Raw sequencing data were filtered using Trimmomatic v 0.38 [22]. Adapters were removed, and low-quality read ends with an average Phred quality score <15 within a 4-bp window were trimmed. Reads with an average Phred quality score <15 and/or shorter than 36 bp after adapter removal and trimming were discarded.

Six isolates were additionally sequenced on the MinION platform (Oxford Nanopore Technology, UK) following a previously published protocol [6].

### 2.3 Assembly-dependent genotyping

Filtered reads were error-corrected and assembled using SPAdes v 3.13.1 [23]. The taxonomic assignment was confirmed using BLAST search against the NCBI RefProk database (release 18.10.2018). AMR genes were detected by nucleotide BLAST search against CARD (release 11.10.2018) [24] and ResFinder (release 22.01.2019) [25]. MLST analysis was performed using PubMLST schemes built into the MLST plug-in of the OmicsBox (BioBam, Spain). Whole genome pairwise alignment was performed with CLC Genomics workbench (Qiagen, Germany). Assemblies were annotated using Prokka [26] and core genome analysis was performed using Roary with 75% minimum protein similarity [27]. MAFFT (Multiple Alignment using Fast Fourier Transform) was used for core genome alignment [28]. Maximum likelihood core genome phylogenetic tree was build using IQ-Tree [29] with default and pangenome matrix was visualized using *roary_plots.py* script from Roary [27].

### 2.4 Assembly-free genotyping

All analyses were performed in MATLAB® v2018 (MathWorks Ltd, USA) unless stated otherwise.

#### 2.4.1 β-lactamase associated k-mers (BLAKs) database development

We extracted all β-lactamase associated genes from CARD v3.0 (release 11.10.2018) [24] and ResFinder (release 22.01.2019) [25], combined them into one collection and removed all the duplicates. This newly generated database contained 1872 unique β-lactamase associated entries. Each gene entry was subsequently transformed into 21-bp k-mers using *kmercountexact.sh* command (BBMap v38.32 [30]) and all unique k-mers (n = 294 636) were extracted. These k-mers will be further referred to as β-lactamase associated k-mers (BLAKs).

#### 2.4.2 Mapping of bacterial genomes to BLAKs database

All filtered reads were transformed into 21-bp k-mers using the *k-mercountexact.sh* command (BBMap v38.32 [30]). To reduce the number of k-mers caused by sequencing error, only k-mers detected in the dataset more than five times were kept (*mincount* = 5). These k-mers were then mapped to the BLAKs database, and sequencing information was transformed into a line with the presence/absence of the BLAKs.

As a result, the dataset was then represented as an *n*-by-*k* binary table where *n* is the number of bacterial isolates, and *k* denotes the number of BLAKs.

#### 2.4.3 Neural network modeling

Feed-forward neural network models for prediction of WT/NWT phenotype (and in cases of S/R phenotype) were built for each bacteria-antibiotic combination using the Deep Learning toolbox for MATLAB® (MathWorks Ltd., USA). Eighty percent of the data were used for training, 10% for validation, and 10% for testing each model.

For each antibiotic, we first selected featured BLAKs, which were differentially represented in WT/NWT strains using Neighboring Component Analysis (NCA). Isolates were partitioned into 5 folds of train and test data. For each fold, NCA with twenty linearly spaced λ values (regularization parameter) between 0 and 0.5 were performed and loss in classification for each test set was calculated. Finally, NCA was performed using optimized λ on all data. A subset of BLAKs with feature weight exceeding threshold was then used as the input for the neural network model. For each model, the threshold was set on the right-end of the features’ weight distribution histogram (Supplementary Fig. 1).

The dataset was split into train, validation and test subsets (80%/10%/10% of data respectively). A set of featured BLAKs was used as an input layer for the neural network (all BLAKs were treated equally and no weights were preset to the NCA weights), whereas the number of hidden layers, as well as the number of neurons in them, varied depending on the size of the input layer. If number of featured BLAKs was below 50, then one-layered network was used. First, a network with one hidden layer and 12 neurons was trained. Then, number of neurons was increased by 12*n and a new network was trained as long as 12*n < number of BLAKs. In case number of BLAKs was above 50, two hidden layers with 24 and 12 neurons in each was trained, and number of neurons in each layer in further iterations was also increased by *n* times. A scaled conjugate gradient backpropagation algorithm was used for weights- and bias values update during model training. Hyperbolic tangent sigmoid (*tansig*) activation function was used between inner layers. The *softmax* classification layer comprising two neurons (WT/NWT and S/R) was used as an output layer. Finally, networks with best prediction rate for each species-antibiotic set was selected.

## 3 Results

### 3.1 Phenotypic characteristics of the dataset

The AMR-Diag dataset comprised 90 strains of *E. coli* and 76 strains of *K. pneumoniae,* both from Norway and India, with varying resistance profile against ampicillin (AMP), cefotaxime (CTX), ceftazidime (TAZ), meropenem (MEM), and imipenem (IMI) (Table 1). Third-generation cephalosporins (CTX, TAZ) had the most even representation of both WT and NWT isolates regardless of species (*E. coli* – 60%/40%; *K. pneumoniae* – 50%/50%), whereas, in the case of carbapenems, WT was overrepresented in our dataset. The majority of NWT isolates were of Indian origin. Detailed information on MIC values, as well as inhibition zone diameter interpretation for ertapenem (ERT), can be found in Supplementary Table A.

**Table 1:** MIC distribution of the isolates. The cut-off between WT (light blue) and NWT (no coloring) is set according to EUCAST ECOFF values [20]. N – number of NWT isolates. **A**. *E. coli*, **B**. *K. pneumoniae*.

### 3.2 Genotypic characteristics of the dataset

#### 3.2.1 WGS stats

The sequencing data comprised 5 403 000 ± 2 693 600 [mean ± SD] raw paired-end reads per isolate, and 95.4% ± 6.2% of them remained after adapter trimming and quality filtering. Overall assemblies comprised 199 (208) [median (IQR)] contigs of ≥ 500 bp length with a total length of 4 997 094 ± 1 648 995 bp (Table 2; Supplementary Tables B and C). Whole genome pairwise identities ranged up to 98.4% for *E. coli* and 99.4% for *K. pneumoniae* (Supplementary Tables D and E). Core genome phylogenetic trees, as well as heatmap of genes presence/absence are provided in Supplementary Fig. 2.

**Table 2:** General statistics of the de novo assemblies.

Species	Number of contigs [median (IQR)]	Largest contig, bp [mean ± stdev]	Total length, bp [mean ± stdev]	GC,% [mean ± stdev]	N50, bp [mean ± stdev]
*E. coli*(n = 90)	199 (208)	412 016 ± 239 231	5 133 633 ± 1 156 150	51 ± 1	157 841 ± 114 466
*K. pneumoniae*(n = 76)	119 (381)	421 222 ± 312 860	4 848 755 ± 2 044 236	57 ± 2	149 514 ± 114 415

#### 3.2.2 Resistome analysis

For *E. coli*, TEM-1 was the most prevalent β-lactamase in Norway, being detected in 69% of Norwegian isolates (Supplementary Table B). TEM-1 was detected in 42% of the Indian isolates also. However, in India, CTX-M-15 (73%) and non-carbapenemase-OXAs (60.0%) were the most frequently detected β-lactamase-encoding genes among the acquired ones. Twenty-one percent of Indian isolates possessed NDM-5 metallo-β-lactamase (MBL), whereas in Norway, NDM was not detected and VIM-1 MBL was found only in one *E. coli* isolate.

As SHV genes are intrinsic in *Klebsiella*, they were the most frequently detected β-lactamase-encoding genes in *K. pneumoniae* (Supplementary Table C). Nearly all of the isolates where SHV was not detected had genome coverage below 50% of the genome size (Supplementary Table C). Prevalence of TEM-1, CTX-M-15, and OXA-232 (OXA-48-like) exceeded 60% of Indian isolates and comprised 5–20% in the Norwegian isolates. Seven Indian isolates possessed NDM-1/5 MBL. None of the Norwegian isolates possessed any carbapenemase encoding genes. Geographical differences between resistome profiles of the isolates can be found in Supplementary Fig. 3.

#### 3.2.3 Multi locus sequence typing

The dataset comprised 37 sequence types of *E. coli* and 31 sequence types of *K. pneumoniae* (Supplementary Table A). The most prevalent sequence types for *E. coli* were ST 131 (13 isolates both from Norway and India), ST 95 (10 isolates from Norway), ST 405 (5 isolates from India), and ST 73 (5 isolates from Norway). For *K. pneumoniae*, the most prevalent STs were ST 147 (8 isolates from India) and ST 231 (7 isolates from India). No correlations between sequence types and isolation sources were observed (Supplementary Table A).

### 3.3 β-lactamase k-mers associated with wild type/non-wild type delineation are drug class and species-specific

**Fig. 1:**
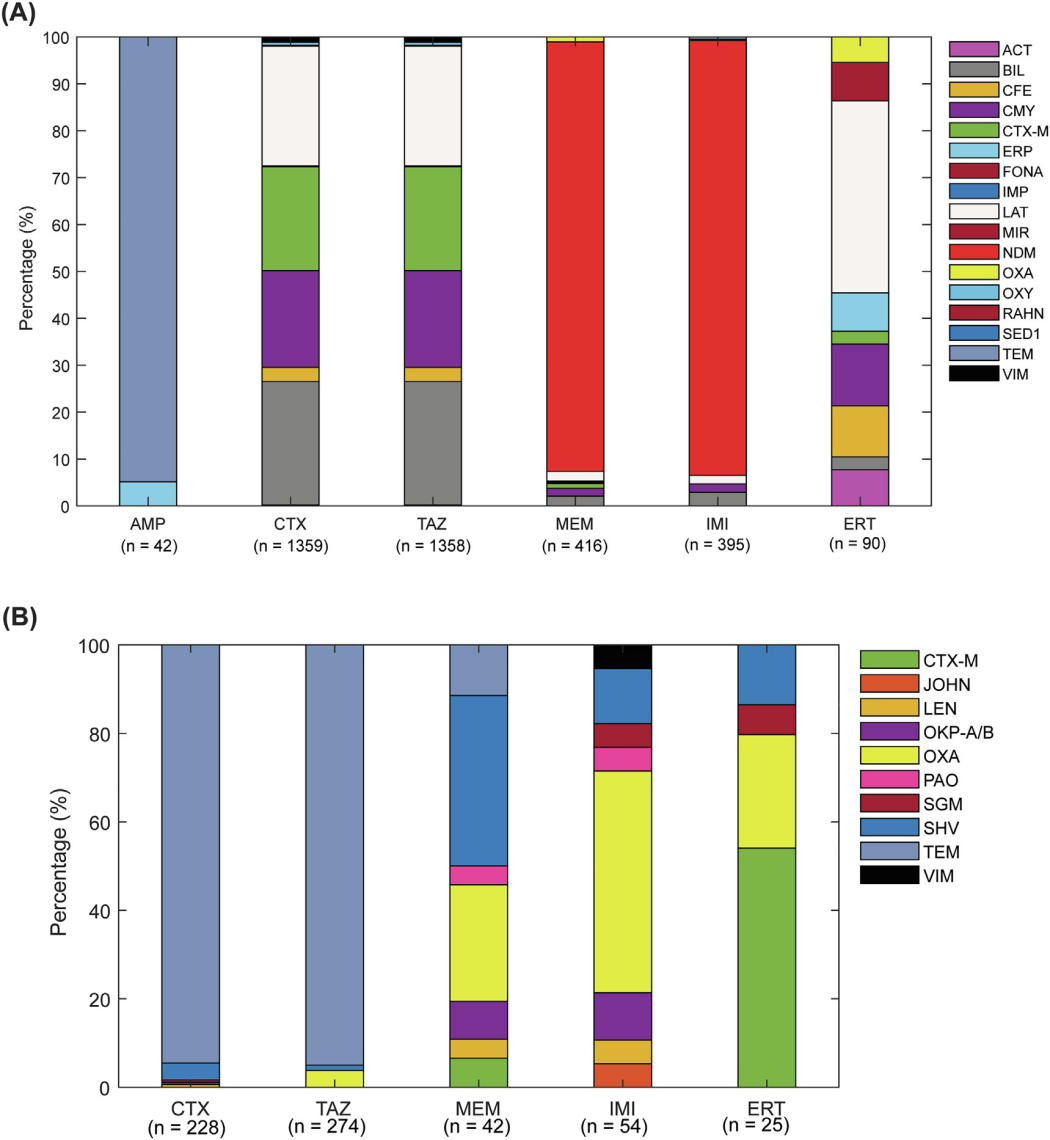
Prevalence of featured BLAKs associated with antibiotic resistance in various gene groups in (A) *E. coli* and (B) *K. pneumoniae.* The number of featured BLAKs in each species-antibiotic pair is given in brackets.

For each species-antibiotic combination, we searched for the BLAKs that were associated with the phenotype using NCA, i.e., featured BLAKs. The number of featured BLAKs varied from 6 to 416, and the majority of these BLAKs were located on NDM, CTX-M, LAT, BIL, CMY gene families in case of *E. coli*, and on TEM, OXA, SHV, and CTX-M gene families in case of *K. pneumoniae* (Fig. 1). Detailed information on each gene variant that has featured BLAKs in its sequence is given in Supplementary Tables F and G.

In *E. coli*, 99.9% BLAKs associated with ceftazidime resistance were also associated with cefotaxime. These BLAKs nearly equally stemmed from CTX-M, BIL, CMY, and LAT genes and covered all the sequences (Supplementary Fig. 4). Thirteen BLAKs associated with both cefotaxime and ceftazidime resistance stemmed from the 3′-end of the VIM-48 gene. The majority of BLAKs that were associated with *E. coli* resistance to meropenem (379 out of 395) were also associated with resistance towards imipenem, and all of them stemmed from NDM and spanned the whole gene (Supplementary Fig. 4). Ertapenem resistance-associated BLAKs originated mostly from the CMY genes, also spanning the entire gene.

For *K. pneumoniae*, 213 BLAKs were commonly associated with 3rd gen. cephalosporin resistance. The majority of them originated from the TEM gene family, covering three distinct regions (Supplementary Fig. 5). Meropenem and imipenem resistance was associated with BLAKs from SHV and OXA, and 30 of these BLAKs were found in common, covering two loci in SHV (Supplementary Fig. 5) and one region of OXA (300 bp-700 bp; Supplementary Fig. 4). Interestingly, although ertapenem resistance-associated BLAKs were also associated with the OXA gene family, all of these BLAKs were located much further downstream (2100–2500 bp region).

Although the TEM-1 gene prevailed AMR-Diag dataset, nearly all of the featured BLAKs that mapped to TEM genes mapped to highly conserved regions of TEM genes since they were found in all 193 TEM variants included in CARD and ResFinder (Supplementary Tables F and G). OXA, CTX-M, and SHV BLAKs were also located in regions found in many, albeit not all, genes belonging to these families. In VIM, however, all featured BLAKs were exclusively located on the 3′-end of the VIM-48 (Supplementary Table F).

### 3.4 Wild type vs. non-wild type phenotype prediction

#### 3.4.1 Prediction of wild type vs non-wild type

Generally, AMR-Diag trained neural networks had higher prediction rates for *K. pneumoniae* than *E. coli* (Table 3). Apart from the *E. coli* – ampicillin prediction network, the overall accuracy varied from 94% to 100%. Ampicillin prediction in *E. coli* was relatively low with an 80% precision (proportion of true NWT in all that are assigned to NWT) and 80% recall (proportion of actual NWT that was correctly assigned) in the test subset of data, therefore we decided to exclude this network from further analysis. For *K. pneumoniae*, we did not attempt to create an ampicillin prediction network since this species is intrinsically resistant towards ampicillin, and only one *K. pneumoniae* isolate was susceptible to it. ROC curves for trained models can be found in Supplementary Figs. 6 and 7 for *E. coli* and *K. pneumoniae* respectively. List of isolates that were used in train/validation/test subsets, as well as their maximum pairwise identity to isolates from other subsets is given in Supplementary Table H.

**Table 3:** Accuracy, precision and recall of 12 feed-forward neural networks for WT/NWT prediction of *E.coli* and *K. pneumoniae* isolates. *HLN – number of neurons in hidden layers.

Bacteria	Antibiotic	HLN*	Number of isolates correct/all Accuracy, %						Precision/Recall for NWT isolates from test subset, %
			Train [80%]		Validate [10%]		Test [10%]		
			WT	NWT	WT	NWT	WT	NWT	
*E. coli*	AMP	24; 12	24/24	40/46	5/6	3/3	3/4	4/5	80/80
			91		89		78		
	CTX	128; 64	44/44	25/26	6/7	2/2	7/7	4/4	100/100
			99		90		**100**		
	TAZ	128; 64	42/43	25/27	6/6	3/3	8/8	3/3	100/100
			96		**100**		**100**		
	MEM	192; 96	55/55	12/15	8/8	1/1	10/10	1/1	100/100
			97		**100**		**100**		
	IMI	48; 24	63/63	3/7	7/7	1/2	8/8	3/3	100/100
			94		89		**100**		
	ERT	24; 12	49/50	19/20	4/4	5/5	8/8	3/3	100/100
			97		**100**		**100**		
*K. pneumoniae*	CTX	96; 48	27/27	33/33	5/5	2/3	5/5	3/3	100/100
			100		87		**100**		
	TAZ	48; 24	25/25	32/34	6/6	2/2	5/5	3/3	100/100
			97		**100**		**100**		
	MEM	24	41/41	18/19	6/6	1/2	6/6	2/2	100/100
			98		87		**100**		
	IMI	24; 12	42/44	15/16	6/6	2/2	5/5	3/3	100/100
			100		**100**		**100**		
	ERT	12	37/37	23/23	3/3	5/5	4/4	4/4	100/100
			100		**100**		**100**		

**Fig. 2:**
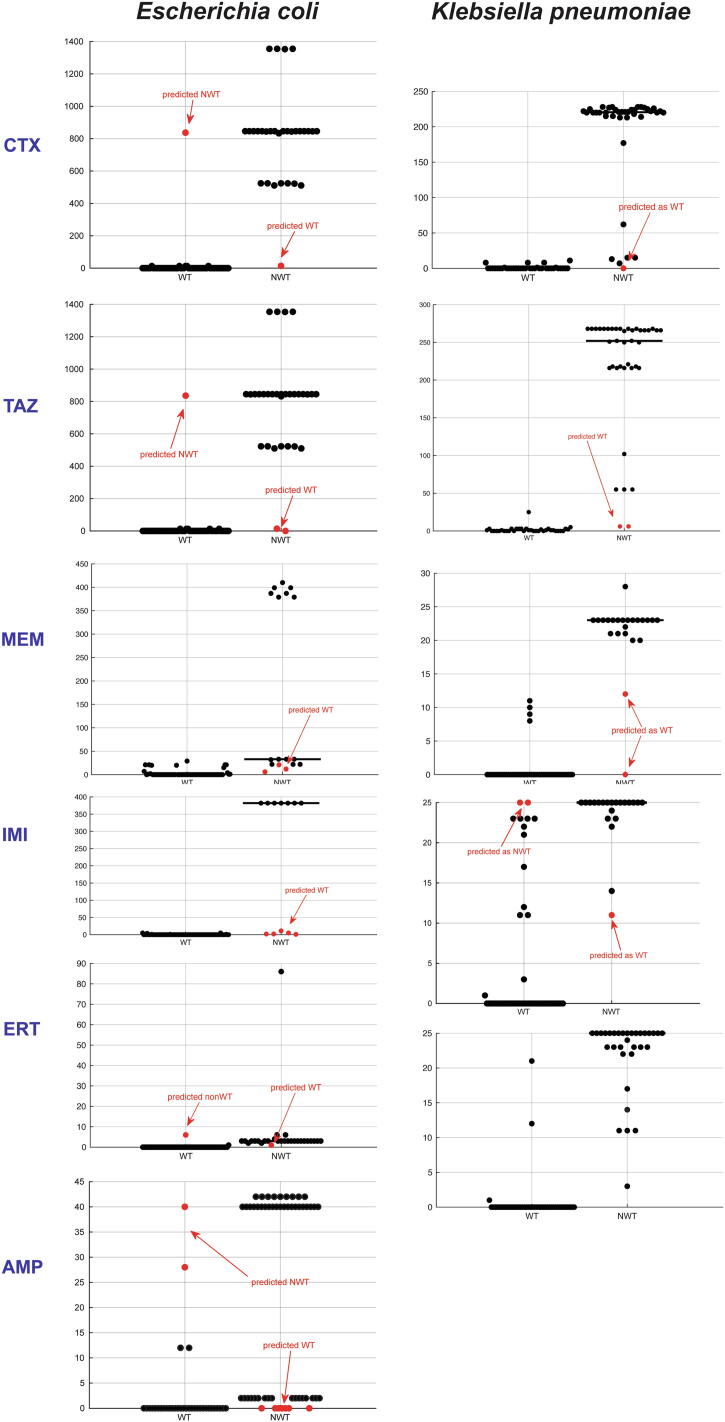
BLAKs distribution in *E. coli* and *K. pneumoniae* isolates for the different *β-lactam* antibiotics. Wild type (WT) and non-wild-type (NWT) isolates.

As expected, the number of featured BLAKs was significantly lower in WT isolates (FDRp < 0.05; Fig. 2). In most cases, those isolates assigned incorrectly on training or validation stages, had as many featured BLAKs as isolates from the other class. Out of three *E. coli* isolates wrongly predicted with regard to 3rd gen cephalosporins WT/NWT, two of them have MIC values close to the ECOFF threshold (Supplementary Table I). It may be highlighted that the majority of wrongly predicted strains with regards to carbapenems had MIC value far off the ECOFF threshold (Supplementary Table I). In addition, all the incorrectly predicted isolates were also checked for efflux pumps, OMP, and point mutations (Supplementary Table I). Mutations in protein binding protein PBP3 (D350N, S357N) were detected in all but one isolates, *ompA* and *ompK37* porin genes were detected in *K. pneumoniae.* Various efflux pumps associated with *β-*lactam resistance were detected in all isolates. We have also attempted to train k-mer based neural networks for colistin resistance prediction. All our isolates were colistin-sensitive, so we downloaded publicly available data for colistin-resistant isolates of *E. coli* and *K. pneumoniae* from the EMBL-EBI European Nucleotide [31] and Macesic et al. [9]. However, this k-mer based approach did not work for colistin resistance prediction (Supplementary Tex 1).

#### 3.4.2 Prediction of S/R phenotypes

Generally, all isolates fell into the ‘S’ or ‘R’ phenotype category based on the EUCAST clinical breakpoints v10.0 (valid from 01.01.2020), so our WT/NWT prediction models could represent ‘S’/‘R’ prediction models. However, there were two strains of *E. coli* that showed ‘I’ phenotype for ceftazidime and for meropenem and four strains of *K. pneumoniae* that exhibited ‘I’ phenotype towards imipenem. Therefore we excluded these strains and created new networks for ‘S’/‘R’ phenotype prediction (Supplementary Table J). The accuracy for ‘S’/‘R’ networks ranged from 91 to 99%, and they had a comparable number of featured BLAKs to the WT/NWT models. The majority of these BLAKs also belonged to the same genes (Supplementary Table K).

### 3.5 Cross-performance of phenotype prediction machine learning algorithms

To test how our networks would perform on external data, we have used previously published datasets. For *K. pneumoniae*, we downloaded 1491 genomes from Nguyen et al. (2018) [11], where authors have trained models to predict MIC values. For *E. coli*, we have downloaded 134 randomly selected genomes from Kim et al. (2020) [10], where authors have trained models for both AMR gene content and phenotype prediction. Additionally, we have tested how these previously published algorithms perform on our isolates.

For *E. coli*, both algorithms tended to under-predict NWT phenotypes from each other’s datasets (Table 4). Both datasets had genes from CTX-M, TEM, and OXA families among the most prevalent ones (Supplementary Table M). However, LAT genes were prevalent in the AMR-Diag dataset, and VAMPr isolates contained KPC genes, which were absent in AMR-Diag.

**Table 4:** Cross-performance of neural networks for prediction of *E. coli* AMR phenotype. Precision/recall for WT and NWT classes are given below the count of isolates.

	TAZ		MEM		IMI		ERT	
	WT	NWT	WT	NWT	WT	NWT	WT	NWT
VAMPr [10] models & AMR-Diag genomic data	51/56	25/30	71/71	7/15	73/73	7/13	55/59	7/17
	91%/91%	83%/83%	90%/100%	47%/47%	92%/100%	54%/93%	85%/93%	64%/41%

AMR-Diag models & VAMPr [10] genomic data	24/38	56/66	90/90	3/10	13/13	0/4	79/79	0/14
	71%/63%	80%/85%	93%/100%	100%/30%	76%/100%	0%/0%	85%/100%	0%/0%

For *K. pneumoniae*, both studies had algorithms for three antibiotics – ceftazidime, meropenem, and imipenem. The networks exhibited dataset-dependent performance (Table 5). AMR-Diag networks missed out on the NWT phenotype of Nguyen et al. [11] isolates, whereas Nguyen et al. [11] models failed to predict WT isolates from our dataset. Same as in our dataset, the most prevalent β-lactamase genes in *K. pneumoniae* isolates from Nguyen et al. belonged to SHV, OXA, TEM, and CTX-M groups (Supplementary Table N). However, unlike our isolates, genomes of *K. pneumoniae* from Nguyen et al. [11] contained KPC. Besides, our isolates had NDM genes that were not detected in Nguyen et al. [11] dataset.

**Table 5:** Cross-performance of ML algorithms and datasets for prediction of *K. pneumoniae* AMR phenotype. Precision/recall for WT and NWT classes are given below the count of isolates.

Models/datasets	TAZ		MEM		IMI	
	WT	NWT	WT	NWT	WT	NWT
Nguyen et al. [11] models & AMR-Diag genomic data	0/36	38/38	0/52	22/22	5/54	16/20
	0%/0%	51%/100%	0%/0%	30%/100%	55%/9%	25%/80%
AMR-Diag models & Nguyen et al. [11] genomic data	7/10	1096/1481	1025/1025	0/352	1000/1088	1/482
	2%/70%	99%/74%	74%/100%	0%/0%	68%/92%	1%/0%

In addition, we have also tested 193 isolates of *K. pneumoniae* (Supplementary Table O) and 126 isolates of *E. coli* (Supplementary Table P) downloaded from the NCBI Pathogen Detection database (https://www.ncbi.nlm.nih.gov/pathogens/). Same as in cross performance tests, Nguyen models tended to over-predict NWT phenotype, whereas AMR-Diag over-predicted WT phenotype (Supplementary Table Q). In case of *E. coli*, both models exhibited comparable performance (Supplementary Table R).

### 3.6 Ensemble networks for prediction of phenotype across different studies improve their performance on unseen data

We also trained separate neural networks for phenotype prediction of 1491 *K. pneumoniae* isolates from Nguyen et al. [11] and combined them with AMR-Diag networks into an ensemble network. In the ensemble, a given isolate was assigned to an NWT class if either network classified it as NWT. Generally, this approach improved the prediction for cross model performance compared to the network alone (Table 6). However, we also observed that in the case of ceftazidime, for AMR-Diag dataset prediction using the ensemble approach, the accuracy dropped to 52%. Also, no ceftazidime WT was predicted in the Nguyen et al. [11] data. This is due to the fact that the network trained on Nguyen et al. [11] data assigned all isolates as NWT. Only 10 out of 1491 isolates used from Nguyen et al. [11] data were susceptible to ceftazidime, which indicates a certain input bias in the training data.

**Table 6:** Performance of neural networks ensemble for prediction of *K. pneumoniae* AMR phenotype. Two networks used in the ensemble are a network trained on data from Nguyen et al. and a network trained on AMR-Diag data. Precision/recall for WT and NWT classes are given below the count of isolates.

Models/datasets	TAZ		MEM		IMI	
	WT	NWT	WT	NWT	WT	NWT
Networks ensemble & Nguyen et al. [11] data	0/10	1481/1481	974/1025	319/352	983/1028	290/322
	0%/0%	100%/99%	97%/95%	86%/91%	97%/96%	86%/90%
Networks ensemble & AMR-Diag data	excluded*		53/53	22/22	55/55	20/20
			100%/100%	100%/100%	100%/100%	100%/100%

### 3.7 AMR-Diag networks are independent of the sequencing platform, sample type and don’t require high genome coverage

We selected three *E. coli,* and three *K. pneumoniae* isolates from Norway and re-sequenced them on the ONT MinION platform using previously published protocol [6]. Phenotypes of all tested *E. coli* isolates were predicted correctly. In the case of *K. pneumoniae*, all except 3/30 antibiotic-bacteria combinations were wrongly predicted, two as ceftazidime-WT and one - as cefotaxime-WT (Supplementary Table S).

In addition, we have also tested the performance of AMR-Diag networks on previously published MinION sequencing data of blood cultures spiked with CTX-M-containing *E. coli* and *K. pneumoniae* [6]. Here we took both partial data, generated up to the point when the first sequence containing target AMR genes (10 – 59 min from the sequencing start), and all data generated throughout the whole sequencing run. Prediction results were identical in both cases (Table 7). Interestingly, already after 40 min of sequencing, *K. pneumoniae* A2-23 was correctly assigned as NWT using our networks. In the case of *E. coli* A2-39, AMR-Diag networks assigned this isolate to a WT with regards to its resistance towards ceftazidime. For this isolate, MIC was measured by disk diffusion test, and its zone diameter was 19 mm, whereas EUCAST WT/NWT cut-off is 21 mm.

**Table 7:** Phenotype prediction of partial ONT MinION data of spiked blood cultures, sequencing data were taken from the sequencing start-up to the time point, where the first target ARG was detected, as well as from the whole sequencing run. Predictions that correspond to the correct phenotypic data are highlighted in bold.

Blood culture spiked with	Time when first ARG was detected	Phenotype prediction (class; score) At the time of ARG detection/all sequence data included				
		CTX	TAZ	MEM	IMI	ERT
*E. coli* A2-39	59 min	WT; 1.0/WT; 1.0	WT; 1.0/WT; 1.0	**WT**; 0.99/**WT**; 0.99	**WT**; 1.0/**WT**; 1.0	**WT**; 0.98/**WT**; 0.99
*K. pneumoniae* A2-23	40 min	**NWT**; 1.0/**NWT**; 1.0	**NWT**; 1.0/**NWT**; 1.0	**WT**; 1.0/**WT**; 1.0	**WT**; 1.0/**WT**; 1.0	**WT**; 1.0/**WT**; 1.0
*K. pneumoniae* A2-37	10 min	WT; 1.0/WT; 1.0	**WT**; 1.0/**WT**; 0.98	**WT**; 1.0/**WT**; 1.0	**WT**; 1.0/**WT**; 1.0	**WT**; 1.0/**WT**; 1.0

Moreover, there were ten isolates with <500 kB raw WGS data (Supplementary Table A). All of these isolates were correctly assigned to a WT class with regards to tested antibiotics. We also tested how *K. pneumoniae*-AMR-Diag networks would perform with closely related *K. quasipneumoniae* species. In 15 out of 18 antibiotic-bacteria combinations, they were correctly assigned to their correct class (Supplementary Table T), which shows that the network could potentially be used for *K. quasipneumoniae*.

### 3.8 Genotype-to-phenotype prediction takes <10 min

Timing tests were performed on a personal laptop with a common configuration (Core i7 CPU, 16 Gb RAM). The k-mer count is done outside of the AMR-Diag and can be performed using any preferred algorithm. We have used the script available from BBMap [30], and in this case, it takes around 3 min per sequence data file. Extraction of featured k-mers and genotype-to-phenotype prediction are performed within the AMR-Diag algorithm, and on average, it also takes about 3 min per file, whereas prediction itself requires only few seconds.

## 4 Discussion

With antibiotic resistance on the rise, we are steadily approaching when we go back to the pre-antibiotic era, where any infection that is now easily curable could become lethal [32]. Globally, antibiotic resistance is not evenly distributed, and there is a large gap between different countries. In a recent report from 2019, Klein et al. have investigated the drug resistance index (DRI) for 41 countries [17]. They have compared the reported data on the use of antibiotics and their resistance for the treatment of infections caused by microorganisms from the WHO priority list (*E. coli*, *K. pneumoniae*, *Acinetobacter baumanii*, *Pseudomonas aeruginosa*, *Staphylococcus aureus*, *Enterococcus faecium,* and *E. faecalis*) [4]. Overall, there was around four-fold difference between India (highest DRI) and Norway (third-lowest DRI). In this study, we report data on isolates both from Norway and India. To our knowledge, this is the first study where these two geographical locations across different ends of the spectrum are included for genotype-to-phenotype prediction.

Different techniques were used for MIC assay in Indian and Norwegian isolates. We did not compare these techniques directly, but in a recent study performed on *Campylobacter* spp.*,* authors compared Sensititre (used in Norway) with E-test strips (used in India) and found concordant results using these two methods [33]. Similar results were reported by Canton et al. in *S. aureus* where they observed good correlation for MRSA determination [34]. Bulik et al. compared the MIC results for E-test, Sensititre and broth dilution assay in *K. pneumoniae* and also found promising concordance between the three, recommending the use of Sensititre and E-test for hospital settings where broth dilution test cannot be performed manually [35].

In this study, there was a very good overlap between BLAKs that were characteristic of phenotypic resistance towards antibiotics of the same class for most cases. In all cases apart from *E. coli* – 3rd gen. cephalosporin models, these BLAKs were characteristic of the whole gene family, indicating that they target conserved regions of these genes rather than specific variant-dependent mutations. In the case of *E. coli* – carbapenem models, the majority of BLAKs spanned the whole sequence of NDM genes (Supplementary Fig. 4), whereas for *K. pneumoniae* – carbapenem models, the majority of BLAKs were located on the transpeptidase domain of the OXA gene family (including both OXA-181 group carbapenemases and OXA-1 group cephalosporinases) (https://www.ebi.ac.uk/interpro/protein/UniProt/P13661/) (Supplementary Fig. 5). BLAKs from the TEM gene family spanned three distinct regions of these genes, including a signal peptide, β-lactamase print, and non-cytoplasmic domain (https://www.ebi.ac.uk/interpro/protein/unreviewed/Q6SJ61/) (Supplementary Fig. 5).

Previous studies [11] have suggested that a logical next step for genotype-to-phenotype prediction will be to train a deeper model to determine if the accuracy of ML methods can be further improved. Moreover, a deep learning method could potentially have more efficient memory usage and reduced computational times [11]. We have built neural networks based on raw unassembled data, which allows rapid phenotype prediction within minutes after the sequencing file is ready.

Further analyses directly from real-time sequencing of blood cultures, rather than the pure culture, would also provide more rapid results but require algorithms for identifying pathogens and eliminating host DNA and other contaminants. In a recently published paper on rapid AMR detection from positive blood cultures [6], we have shown that the use of MinION sequencing can reduce the time needed for bacteria identification and AMR detection to less than four hours from the time when blood culture is flagged positive. Here we have tested AMR-Diag networks on a whole run MinION data from this publication [6] and on partial data that corresponded to the first detected ARG and ranged from 10 to 59 min from the sequencing start. Interestingly, prediction rates were independent of the sequencing data amount as long as one ARG was detected. We have also previously shown that in most cases, it takes around 6–8 h of MinION sequencing to cover 99.9% of the genome at least once. Given this estimate, we speculate that using AMR-Diag networks coupled with MinION sequencing of blood cultures, AMR-Diag networks can predict phenotype as fast as around 1 to 6–8 h from the sequencing start, i.e., 4–11 h after the blood culture is flagged positive.

It has also been previously suggested that error rates of ONT may be too high for effective MIC prediction with an ML method, and it would need to either incorporate an error correction model for processing MinION data or regenerate the model using genomes sequenced with nanopore sequencing [11]. Although AMR-Diag networks were trained using Illumina data solely, they performed well with ONT MinION generated data showing the flexibility of the method and possibility of direct use of MinION reads without any specific preprocessing. However, few MinION-sequenced strains were classified wrong, and it would be interesting to address whether incorporation of error-correcting algorithms prior to phenotype prediction would increase sensitivity. Moreover, for some beta-lactamases (f.ex. SHV, OXA), a single point mutation can completely modify the phenotype [36], [37]. Therefore, a low coverage approach with a relatively high error rate might present a high risk of interpretation mistakes. This limitation should in future be addressed with additional experiments on ESBL *E. coli* or *K. pneumoniae* negative for CTX-M enzymes and with different carbapenemase-producing strains.

Recently, ResFinder [25] has been updated enabling researchers to search for antibiotic resistance genes using raw unassembled data. They also now provide phenotype prediction based on the detected ARGs [38]. In our data, for example, isolates E12 and E19 possessed OXA-181 gene and were thus assumed resistant towards imipenem by ResFinder. These isolates, however, were sensitive towards this drug. AMR-Diag network, on the other hand, correctly classified the isolate as WT (S). Isolates K21 and E15 did not possess ARGs for resistance towards meropenem according to ResFinder, although they were phenotypically resistant and predicted as NWT by AMR-Diag networks. Unlike search for ARGs, AMR-Diag networks are based not on the gene detection per se, but rather on detection of a few 21 bp k-mers from a combination of various ARGs. These examples highlight the usefulness of ML approaches for phenotypic prediction over more simple techniques that focus on resistance gene detection.

To assess the AMR-Diag networks' performance with regards to previously published ML models, we have used data from VAMPr [10] and Nguyen et al. [11] and cross-checked how the models would perform on the isolates from a different dataset. Generally, all models, including AMR-Diag, tended to have lower accuracy for the unseen data as compared to their respective datasets. The reason for that lies most likely in different antibiotic resistance mechanisms that are presented in different datasets. For example, the most notable difference was seen in the carbapenemase resistance genes of *K. pneumoniae*. Nguyen et al. *K. pneumoniae* isolate contained the KPC gene, whereas, in AMR-Diag, NDM genes were present. Since both of these genes are paramount for carbapenem resistance, we decided to train a separate network based on Nguyen et al. data and ensemble with AMR-Diag trained models. This approach largely increased the overall accuracy for both datasets. Given the abundance of various resistance mechanisms and scarcity of sequencing data of phenotypically characterized bacterial isolates, building a universal phenotype prediction network is still a big challenge. However, we believe that this is not an impossible task, and it can be done when more and diverse genotypic and phenotypic linked data becomes publicly available. Medical data are also prone to bias problem due to unequal number of samples in each class [39]. Therefore, ideal dataset should probably include even number of representatives of a) various resistance mechanisms and b) various genotypes of isolates that are susceptible to the given drug. Until then, we believe that the employment of several carefully trained models that cover various mechanisms in the ensemble could be a good option for more accurate phenotype prediction. Since resistance towards β-lactams can also be caused by higher activity of efflux pumps or loss of porins [40], inclusion of these mechanisms to the prediction model may also improve the discussed models. Another limitation of the AMR-Diag approach and other approaches based on in-built databases is its lack of plasticity with regards to updates from CARD and ResFinder. Therefore, exploring self-retraining systems capable of adding new features once ARG database updates are available would also be a big step towards faster and more reliable AST prediction.

## 5 Conclusion

In this work, we have trained neural networks for phenotype prediction of AST from genomic data of *E. coli,* and *K. pneumoniae* isolates from blood, urine, pus, sputum, and other biological fluids of Norwegian and Indian patients. Apart from ampicillin resistance, networks exhibited overall accuracy from 94% to 100% and were capturing resistance mostly caused by TEM, OXA, CTX-M, and NDM genes. Comparison of AMR-Diag networks to previously published genotype to phenotype prediction algorithms that target other mechanisms revealed dataset-dependent performance of all algorithms. However, the accuracy for cross-datasets was increased when they were used in the ensemble. In this work, we also demonstrate the successful use of AMR-Diag networks with ONT MinION data, both from the whole sequencing run of bacterial isolates and from a subset of sequencing data of spiked blood cultures (from the sequencing start to the first output file where the target ARGs were detected). This approach allows genotype-to-phenotype prediction as fast as around 1–8 h from the sequencing start.

## 6 Data availability

The AMR-Diag isolates genomic data, and phenotypic information used during the current study is available from the corresponding author on request. The AMR-Diag networks and algorithms needed to use them can be accessed at https://github.com/rafiahmad-lab/AMR-Diag.

## Authors contributions

R.A., P.K, U.N., K.P., A.K., conceived the study; A.M.T., A.K., and P.S. collected clinical isolates; P.S. and S.A.F. performed the experimental work; E.A. performed data analysis in discussion with R.A. and K.P.; E.A. and R.A. drafted the manuscript. All authors participated in the design of the experiment and edited the manuscript. All authors agree with the final version of the manuscript.

## Declaration of Competing Interest

The authors declare that they have no known competing financial interests or personal relationships that could have appeared to influence the work reported in this paper.
