# Supplementary material for: AMR-Diag: Neural network based genotype-to-phenotype prediction of resistance towards β-lactams in Escherichia coli and Klebsiella pneumoniae
Source: Comput Struct Biotechnol J. 2021 Mar 29;19:1896–906. doi: 10.1016/j.csbj.2021.03.027 (PMC8060595; doi:10.1016/j.csbj.2021.03.027)
Supplement: Supplementary data 1 [file mmc1.pdf]

Supplementary Text A. Neural network training for colistin resistance prediction

In the last 15 or so years, colistin is being used increasingly as a 'last-line' therapeutic to treat infections caused by multidrug-resistant (MDR) Gram-negative bacteria, including *E. coli* and *K. pneumoniae*, when practically no β-lactam options are available [1]. Therefore it is also very relevant to study colistin together with β-lactams, especially 3rd generation cephalosporins and carbapenems.

In Norway, minimum inhibitory concentration (MIC) for colistin was determined by broth microdilution using Sensititre surveillance EUVSEC™ 96 well plates (ThermoFisher). Plates were inoculated using the Sensititre AutoInoculator™ (AIM, V3020, Sensititre), incubated for 18-24 hours, and subsequently read both with the naked eye using the Sensititre Manual Viewbox™ (V4007, Sensititre) and using the Sensititre Vizion Digital MIC Viewing System™ (V2021, Sensititre). *E. coli* ATCC 25922 and *E. coli* IP2.1 were used as quality strains for *E. coli* and *K. pneumoniae* isolates, respectively. In India, MIC to colistin was determined by broth dilution method as per CLSI guidelines [2]. *E.coli* ATCC 25922 was used as a quality control strain.

None of the AMR-Diag isolates exhibited resistance towards colistin. Therefore we obtained genomes of 30 colistin-resistant isolates from the EMBL-EBI European Nucleotide Archive [3] (Supplementary Table J). Data on the MIC values of these isolates were taken from the NCBI Biosample Antibiograms. Also, we have downloaded 164 polymyxin-resistant isolates from Macesic et al. [4]. We downloaded all acquired polymixin resistance genes from ResFinder (n = 54) and have made a list of polymyxin-associated k-mers (n = 20 882) following the same procedure as for BLAKs. However, only one of the polymyxin k-mers had a higher feature weight, whereas all others had the same low feature weight. A BLAST search of EMBL-EBI colistin-resistant isolates against the ResFinder & CARD database revealed the absence of acquired colistin resistance genes. In contrast, there were point mutations in efflux pumps and porins (Supplementary Table I).

Macesic et al. [4] also have attempted to train a machine-learning algorithm to predict polymyxin resistance in *K. pneumoniae* based on k-mers. The authors used k-mers from the whole genome and have concluded that the assembly-based approach worked better for the task. We attempted to build a neural network based only on k-mers from polymyxin resistance genes in this work. However, we did not succeed in training such a network. In *K. pneumoniae*, alterations in chromosomal genes such as *mgrB*, *pmrA/B*, *phoPQ,* and *crrA/B* systems mostly

contribute to the colistin-resistance phenotype [5]. These chromosomal alterations are only partially recognized by ResFinder and CARD databases, which can probably explain a failure to train k-mer based neural networks. For this reason, a different search approach should be carried out.

Reference:

1. Nation, R.L. and J. Li, *Colistin in the 21st century.* Curr Opin Infect Dis, 2009. **22**(6): p. 535-43.
2. CLSI, *Methods for dilution antimicrobial susceptibility tests for bacteria that grow aerobically; approved standard. CLSI document M7-A7.* Clinical and Laboratory Standards Institute, Wayne, PA.
3. Leinonen, R., et al., *The European Nucleotide Archive.* Nucleic Acids Res, 2011. **39**(Database issue): p. D28-31.
4. Macesic, N., et al., *Predicting Phenotypic Polymyxin Resistance in Klebsiella pneumoniae through Machine Learning Analysis of Genomic Data.* mSystems, 2020. **5**(3).
5. Palmieri, M., et al., *Genomic Epidemiology of Carbapenem- and Colistin-Resistant Klebsiella pneumoniae Isolates From Serbia: Predominance of ST101 Strains Carrying a Novel OXA-48 Plasmid.* Front Microbiol, 2020. **11**: p. 294.
