# Supplementary material for: AMR-Diag: Neural network based genotype-to-phenotype prediction of resistance towards β-lactams in Escherichia coli and Klebsiella pneumoniae
Source: Comput Struct Biotechnol J. 2021 Mar 29;19:1896–906. doi: 10.1016/j.csbj.2021.03.027 (PMC8060595; doi:10.1016/j.csbj.2021.03.027)
Supplement: Supplementary data 2 [file mmc2.docx]

*
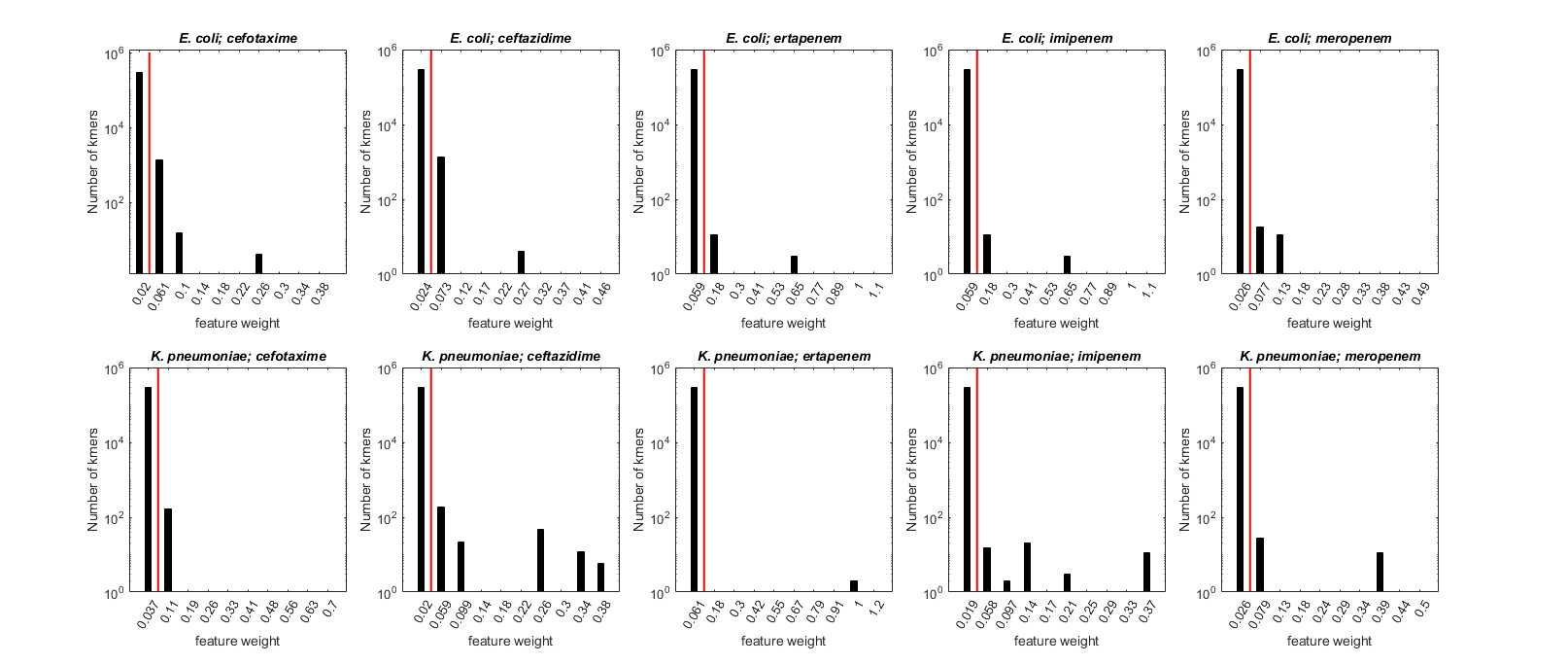
*

Supplementary Figure 1. Cutoff value for featured BLAKs selection

Supplementary Figure 2. Maximum likelihood core genome alignment trees of (A) E. coli and (B) K.pneumoniae.

**A**

| *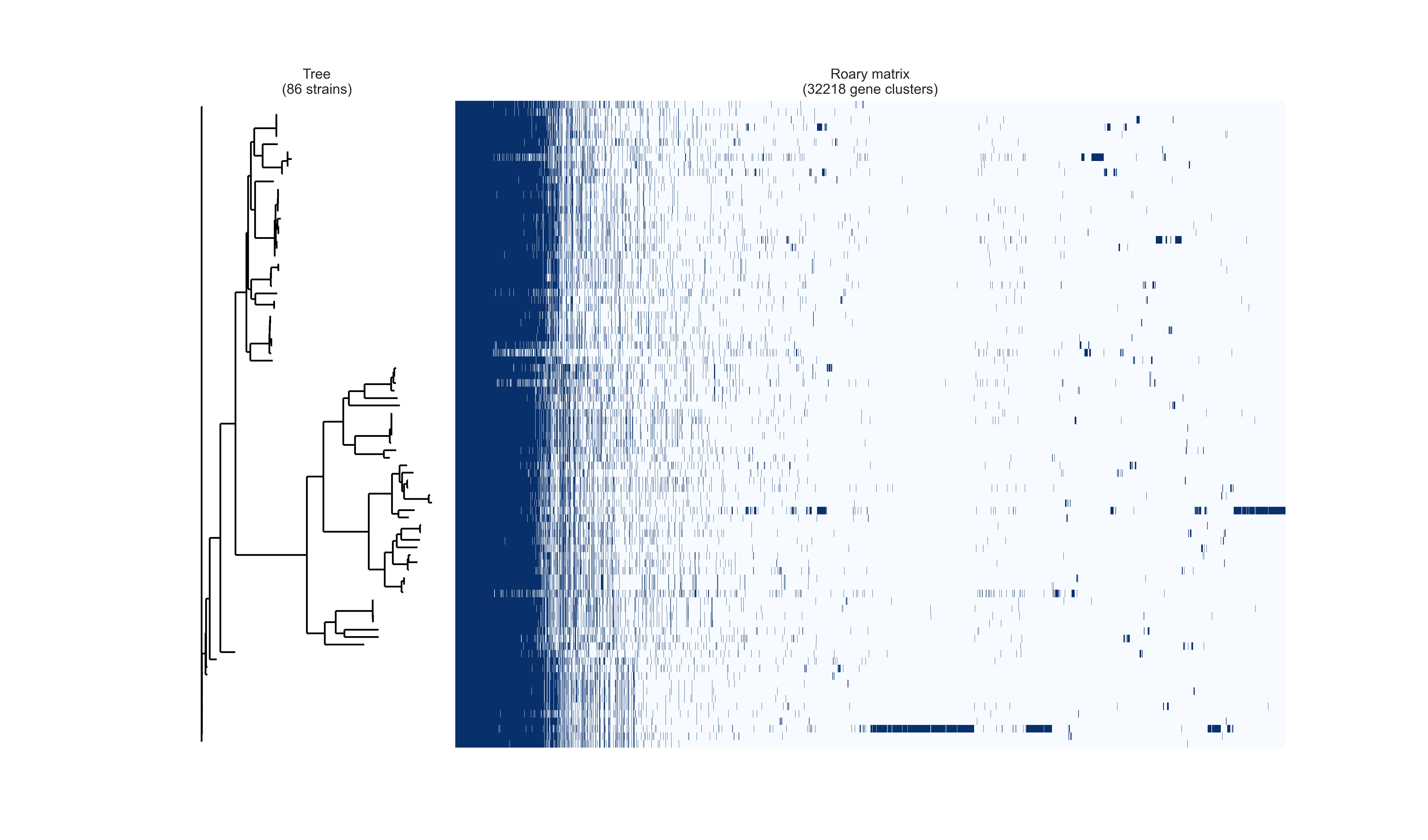* | *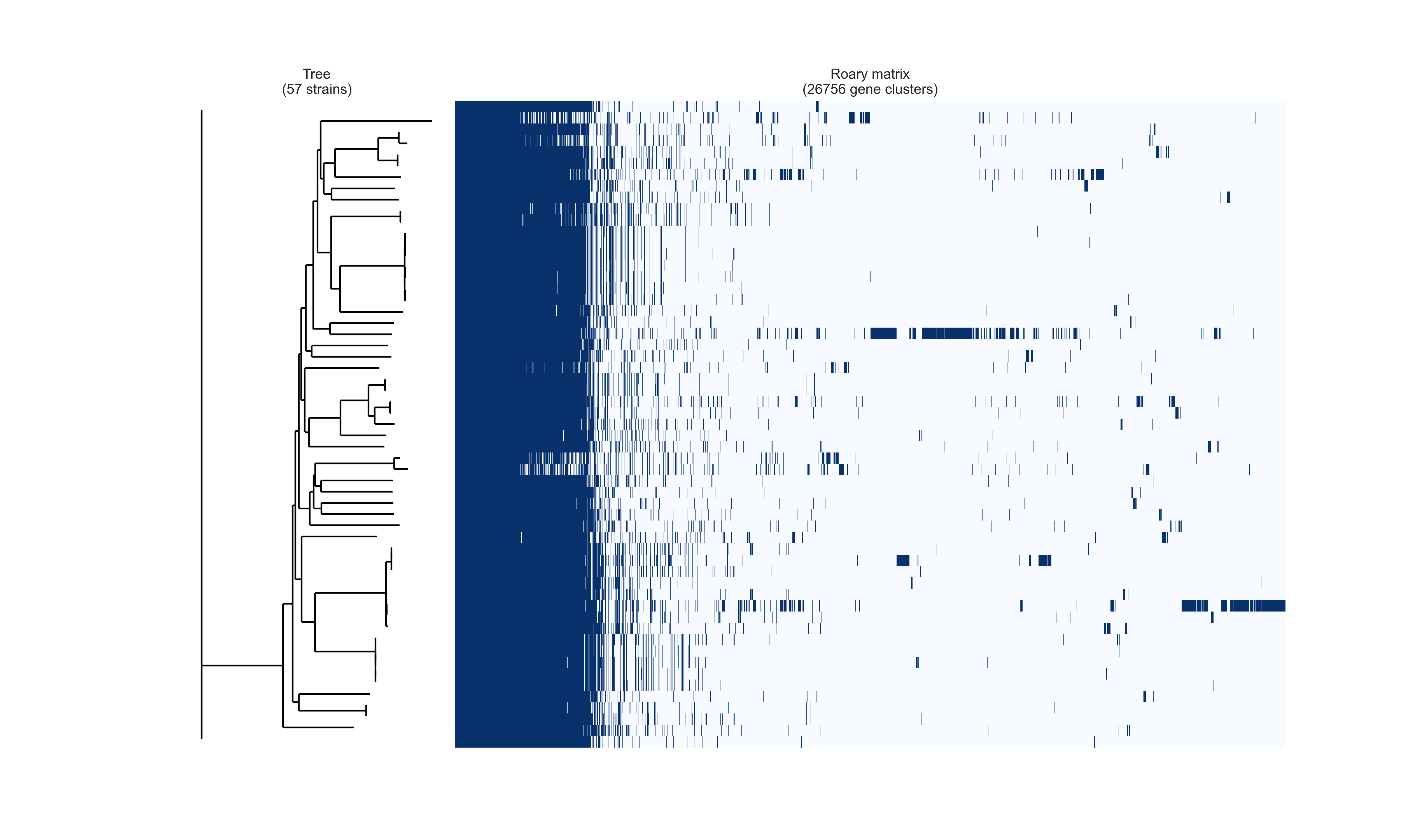*  **B** |
| --- | --- |

Supplementary Figure 3. Prevalence of β-lactamase genes in (A) E. coli and (B) K. pneumoniae isolates from Norway and India based on ResFinder database.

| 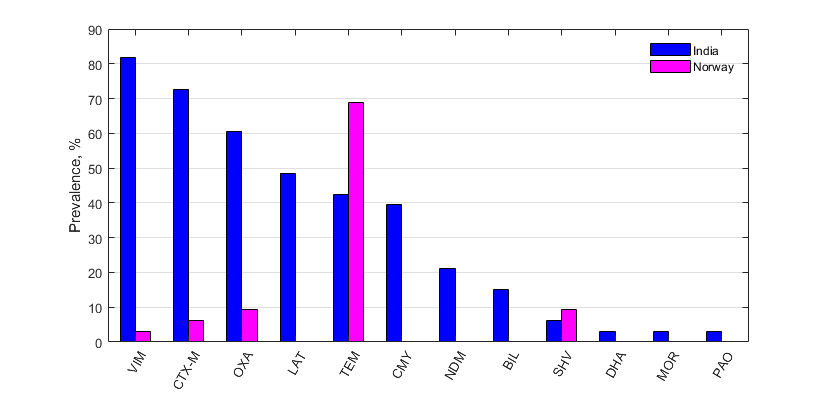  **A** |
| --- |
| 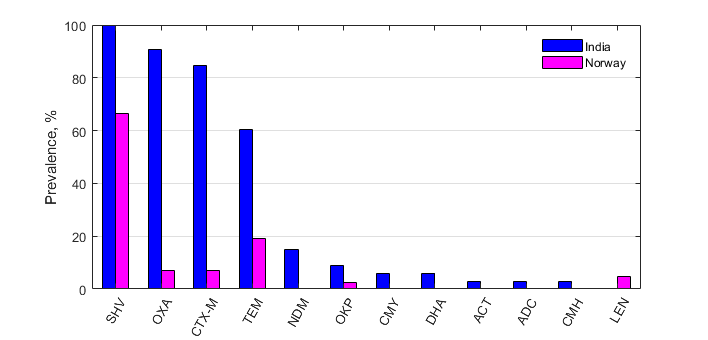  **B** |

| Supplementary Figure 4. Location of Escherichia coli featured resistance-associated BLAKs that were shared between antibiotics on the target genes. | |
| --- | --- |
| 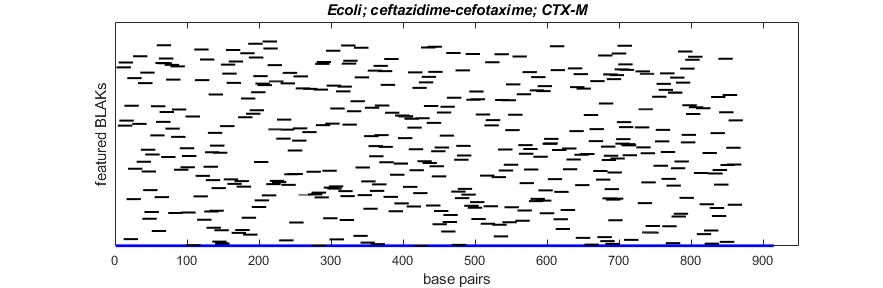 | 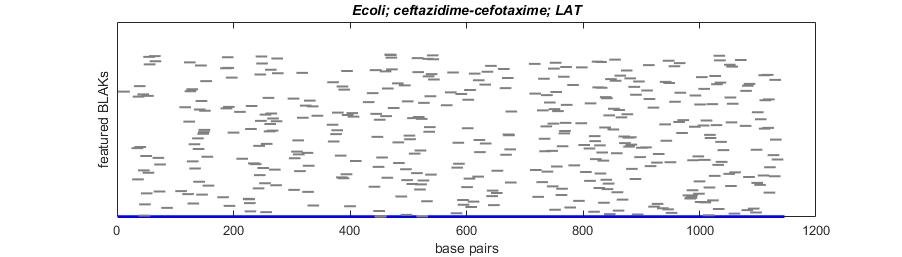 |
| 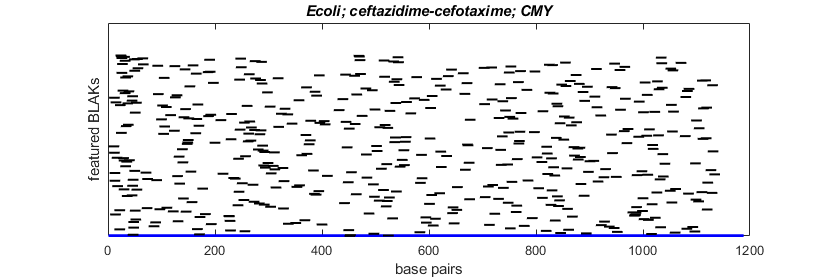 | 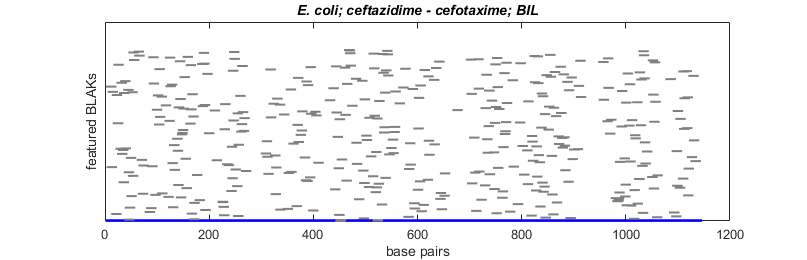 |
| 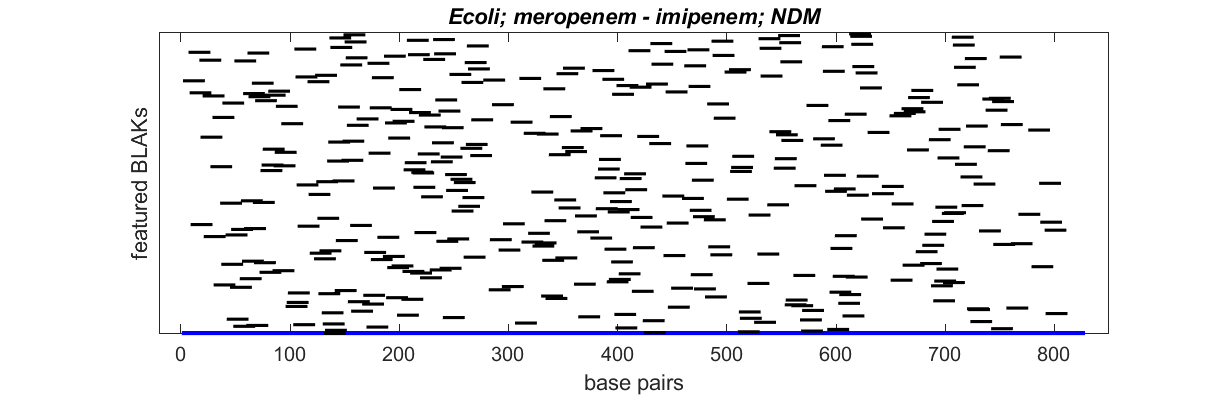 | 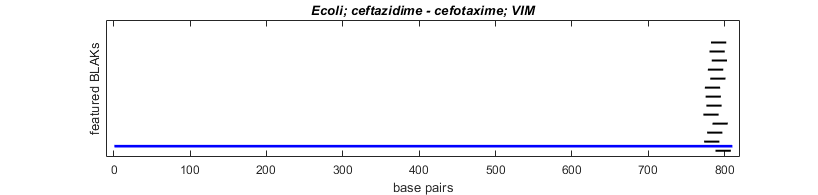 |

| Supplementary Figure 5. Location of Klebsiella pneumoniae featured resistance-associated BLAKs that were shared between antibiotics on the target genes. | |
| --- | --- |
| 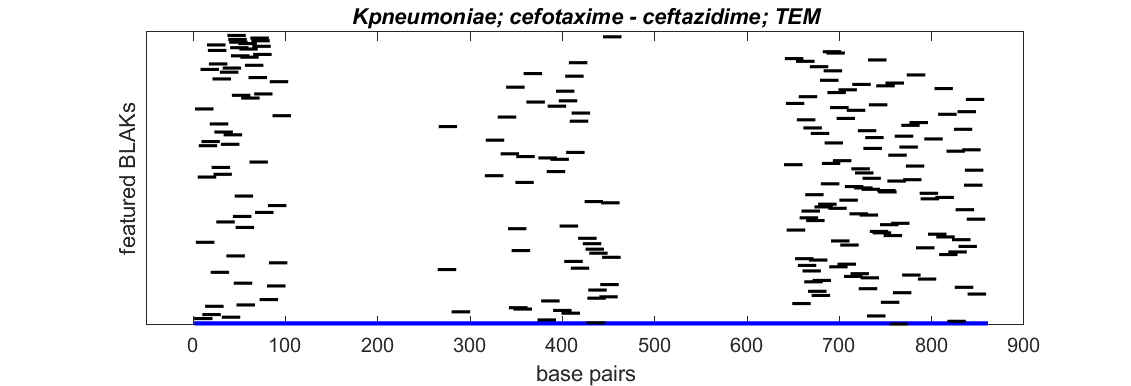 |  |
| 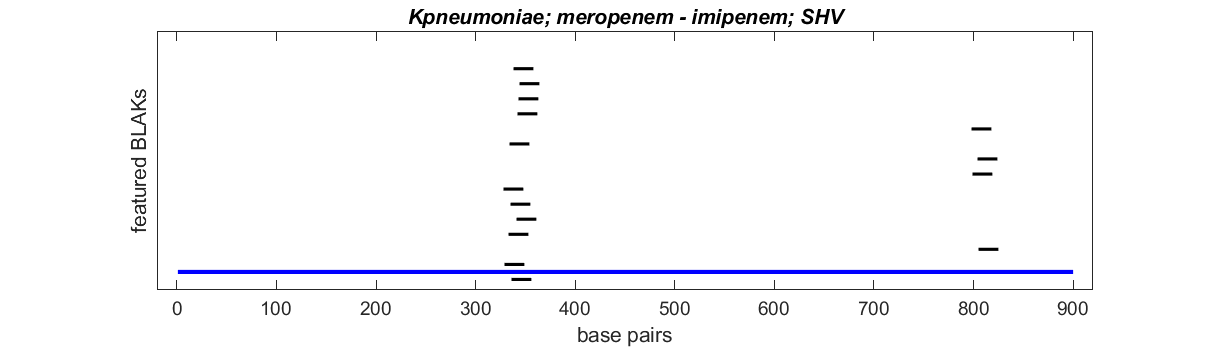 | 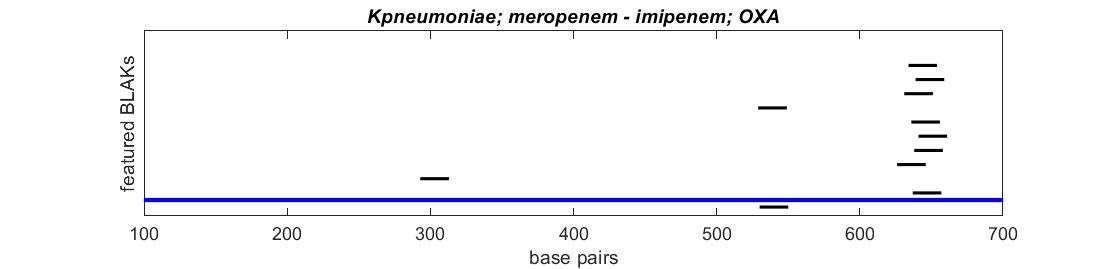 |

*Supplementary Figure 6. ROC curves for E. coli neural networks. Class 1 – WT; Class 2 – NWT.*


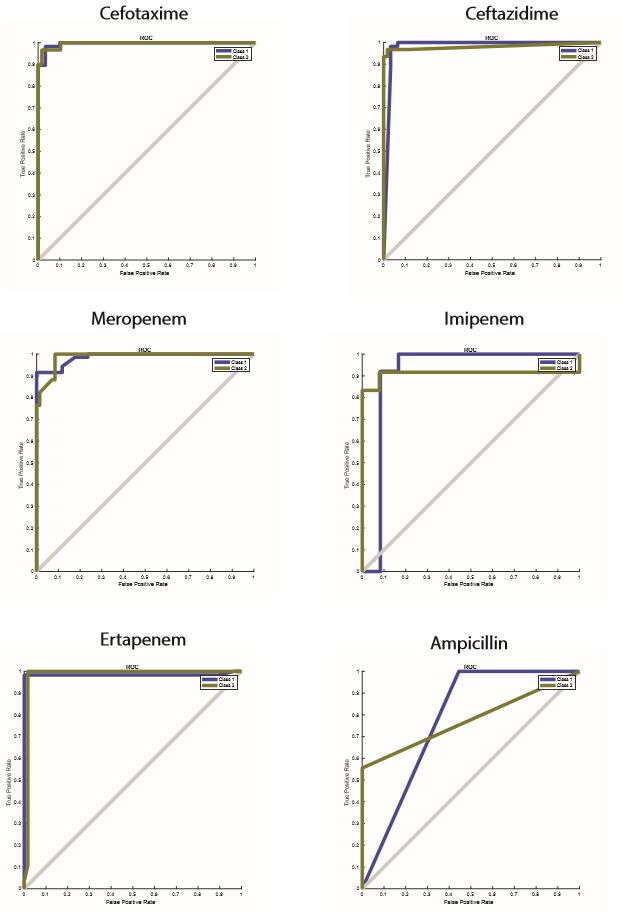


*Supplementary Figure 7. ROC curves for K. pneumoniae neural networks. Class 1 – WT; Class 2 – NWT.*
